# Reducing behavior problems in children born after an unintended pregnancy: the generation R study

**DOI:** 10.1007/s00127-024-02693-3

**Published:** 2024-05-31

**Authors:** Clair A. Enthoven, Jeremy A. Labrecque, M. Elisabeth Koopman-Verhoeff, Mijke P. Lambregtse-van den Berg, Manon H.J. Hillegers, Hanan El Marroun, Pauline W. Jansen

**Affiliations:** 1 Department of Psychology, Education and Child Studies, Erasmus School of Social and Behavioral Sciences, Erasmus University, Mandeville Building, Floor T13, Rotterdam, The Netherlands; 2 Department of Child and Adolescent Psychiatry/Psychology, Erasmus University Medical Center-Sophia Childrens Hospital, Rotterdam, The Netherlands; 3 The Generation R Study Group, Erasmus University Medical Center, Rotterdam, The Netherlands; 4 Department of Epidemiology, Erasmus University Medical Center, Rotterdam, The Netherlands; 5 Simches Division of Child and Adolescent Psychiatry, McLean Hospital and Harvard Medical School, Belmont, MA USA; 6 Department of Psychiatry, Erasmus University Medical Center, Rotterdam, Zuid-Holland The Netherlands

**Keywords:** Unintended pregnancy, Postnatal depression, Social support, Problem behavior, Hypothetical intervention

## Abstract

**Objectives:**

To examine differences in behavior problems between children from intended versus unintended pregnancies, and to estimate how much the difference in problem behavior would be reduced if postnatal depression was eliminated and social support was increased within 6 months after birth.

**Methods:**

Data from the Generation R Study were used, a population-based birth cohort in Rotterdam, the Netherlands (*N* = 9621). Differences in child internalizing and externalizing behavior at ages 1.5, 3, 6, 9 and 13 years between pregnancy intention groups were estimated using linear regression. Associations of postnatal depression and social support with internalizing and externalizing problems were also estimated using linear regression. Child behavior outcomes where compared before and after modelling a situation in which none of the mothers experienced a postnatal depression and all mother experienced high social support.

**Results:**

Most pregnancies (72.9%) were planned, 14.8% were unplanned and wanted, 10.8% were unplanned with initially ambivalent feelings and 1.5% with prolonged ambivalent feelings. Children from unplanned pregnancies had more internalizing and externalizing problems at all ages as compared to children from a planned pregnancy, especially when ambivalent feelings were present. Hypothetically eliminating on postnatal depression reduced the differences in internalizing and externalizing problems by 0.02 to 0.16 standard deviation. Hypothetically increasing social support did not significantly reduce the difference in internalizing and externalizing problems.

**Conclusions:**

Children from an unplanned pregnancy have more behavior problems, in particular when mothers had prolonged ambivalent feelings. Eliminating postnatal depression may help to reduce the inequality in child behavior related to pregnancy intention.

**Supplementary Information:**

The online version contains supplementary material available at 10.1007/s00127-024-02693-3.

## Introduction

Unintended pregnancies are very common all around the world. The highest rates of unintended pregnancies have been found in Latin America and the Caribbean (56%), whereas lower rates are reported in Western Europe (34%) [1]. The Netherlands has a relatively low rate of unintended pregnancies as compared to other countries, yet 18% of the pregnancies are unintended [2]. Whether a pregnancy is unintended or unplanned is complex to determine as feelings towards the pregnancy may change over time [3]. In this study, pregnant women reported during the first trimester of the pregnancy whether the pregnancy was planned or not, and in case of an unplanned pregnancy, how they felt about the pregnancy.

Unintended pregnancies are associated with poorer mental health in children. Research has shown that children born from an unintended pregnancy are more likely to display behavior problems at 5 and 7 years [4], aggression and externalizing problems at 14 years [5], and a lower self-esteem and more depressive symptoms in their 20s [6, 7]. A causal relationship is debatable, as many maternal factors (e.g. lower socioeconomic position and history of psychopathology) that are associated with later child problem behavior may already be present prior to the unintended pregnancy [8]. However, even though potentially explained by other underlying factors, children born from an unintended pregnancy still have a different starting point in life as compared to children from an intended pregnancy.

Knowledge on which pathways drive the difference in child behavioral outcomes of intended versus unintended pregnancies is largely lacking. Two potential pathways are via maternal psychiatric problems (e.g. postnatal depression) and psychosocial problems (e.g. lack of social support), because they are both more common among women with unintended pregnancies [9, 10], are associated with child problem behavior [11] and thus may mediate the association between unintended pregnancy and child problem behavior [12]. Ideally, one would study these pathways using randomized controlled trials, but it is impossible to randomize women into intended versus unintended pregnancies. Therefore, our aim is to study these pathways by modelling a situation in which postnatal depression would be eliminated and social support would be increased using an observational population-based birth cohort [13].

In this study, we examined differences in child behavior between children born from an intended versus unintended pregnancy, and aim to identify mechanisms contributing to these differences. We used a statistical technique called G-estimation to determine the difference in problem behavior across childhood and early adolescence that is associated with (un)intended pregnancies. Then, we estimated how much the difference in child problem behavior would be reduced if postnatal depression symptoms would be reduced and social support would be increased. Our hypothesis is that the absence of postnatal depression and the presence of high social support reduces differences in child problem behavior resulting from (un)intended pregnancies.

## Methods

### Study population

This study is embedded in the Generation R Study, a multiethnic population-based prospective cohort from fetal life onwards. The study is designed to identify early environmental and genetic determinants of growth, development, and health. The cohort has previously been described in detail [14, 15]. Briefly, all pregnant women who resided in Rotterdam at the time of childbirth and with a due date between April 2002 and January 2006 were invited to participate. Enrollment was aimed in early pregnancy (gestational age < 18 weeks), but was possible until birth of the child. In total, 9778 (response rate 61%) women were enrolled in Generation R, of whom 9621 were included in the analyses, see paragraph below. The study has been approved by the Medical Ethics Committee of Erasmus MC in Rotterdam, the Netherlands, and is conducted in accordance with the Declaration of Helsinki of the World Medical Association. Written informed consent was obtained from all parents and children (if older than 12). This project was pre-registered at Open Science Framework (10.17605/OSF.IO/G86SF).

### Measurements

#### Unintended pregnancy

Pregnancy intention was measured using a self-report questionnaire at inclusion of the study. Women reported whether their pregnancy was planned or not. This variable was compared with pre-pregnancy folic acid use and contraceptive use at conception. We excluded those who reported both an unplanned pregnancy and pre-pregnancy folic acid intake (*n* = 104) and those who reported a planned pregnancy and contraceptive use at conception (*n* = 173), leaving *n* = 9621 participants for analyses (*n* = 1765 participants with missing data on pregnancy intention were imputed). In case of an unplanned pregnancy, women reported how they felt about the pregnancy using the following four answering categories: “pleased from the start”; “initially mixed feelings”; “still mixed feelings”; or “mostly unhappy about the pregnancy”. Few women reported having “still mixed feelings” or “mostly unhappy”, which we combined into prolonged ambivalent feelings. Pregnancy intention was categorized into “planned”, “unplanned and wanted from the start”, “unplanned and initially ambivalent feelings”, and “unplanned and prolonged ambivalent feelings”.

#### Child internalizing and externalizing problem behavior

The validated Child Behavior Checklist (CBCL/1½–5 and CBCL/6–18) was completed by the main caregiver of the child at ages of 1.5, 3, 6, 9 and 13 years [16, 17]. The CBCL consists of 99 items (CBCL/1½–5) or 118 items (CBCL/6–18) and assesses behavioral and emotional problems in the preceding 2 (CBCL/1½–5) or 6 (CBCL/6–18) months. Items of the CBCL/1½–5 and CBCL/6–18 were comparable and scored on a 3-point Likert scale (0 = not true, 1 = somewhat or sometimes true, and 2 = very true or often true). Mean scale scores for internalizing (anxious/depressed, withdrawn/depressed and somatic complaints) and externalizing (rule-breaking behavior, and aggressive behavior) behavior were computed and converted to standardized T-scores per wave. Cronbach’s alpha at the different time points were calculated and are shown in supplemental Table 1. Sensitivity analyses were performed with similar scores, but based on self-report by the children. At age 9 years, children were asked to complete the Brief Problem Monitor which is a validated, 19-item abbreviated version of the Youth Self Report (YSR) [18]. At age 13 years, children completed the full YSR containing 110 items [19]. For post-hoc analyses, internalizing and externalizing problem behavior, as measured using the CBCL, were dichotomized based on borderline clinical cutoffs as indicated by a T score of 60 or higher (equivalent to the 84th percentile) as recommended by Achenbach et al. (2000 and 2001) [16, 17]. At ages 1.5, 3 and 6 years, borderline clinical cases were defined as sumscores > 12 for internalizing and > 18 for externalizing behavior. At age 9, borderline clinical cases were defined as sumscores > 8 for internalizling behavior in boys and > 10 in girls, and > 11 for externalizing behavior in boys and girls. At age 13, borderline clinical cases were defined as sumscores > 10 for internalizing behavior in boys, and > 11 in girls, and > 13 for externalzing behavior in boys, and > 11 in girls.

#### Postpartum psychiatric symptoms

Postpartum psychiatric symptoms were measured 2 months after childbirth with the Edinburgh Postnatal Depression Scale (EPDS). This validated self-report questionnaire includes 10 items assessing symptoms of postpartum depression in the previous week, rated on a 4-point scale from 0 (no, not at all) to 3 (yes, quite often) [20, 21]. A sum score ranging from 0 to 30 was calculated with higher scores indicating more depressive symptoms. Women with a score of more than 12 were classified as having postpartum depression. This cut-off score had a sensitivity of over 80% and specificity of 95% in diagnosing postnatal depression in a clinical population [22, 23].

#### Social support

Social support was measured 6 months after childbirth using the Social Support List 12 – Interactions (SSL12-I), a validated 12-item abbreviated version of the full Social Support List – Interactions [24, 25]. Women were asked to indicate whether people around them are nice to them (e.g. give them compliments, invite them to join a party or dinner) or would help them (e.g. provide help in case of illness or moving houses, provide good advice), rated on a 4-point scale from 1 (seldom or never) to 4 (very often). A sum score ranging from 12 to 48 was calculated with higher scores indicating more social support. Women in the lowest tertile were classified as having low social support.

#### Covariates

The following variables were included as covariates and were measured during pregnancy or at birth: maternal age, migration background, socioeconomic factors (maternal education and household income), marital status, maternal and paternal lifetime psychopathology, substance use (drugs, smoking, alcohol) and gestational age at birth [26–29].

### Statistical analysis

Prior to analyses, multiple imputations were performed to replace the missing values in child behavior, unintended pregnancy, postnatal depression, social support and the covariates using MICE package [30]. We created 30 imputed datasets with 100 iterations. Performing multiple imputation on the variables of interest is recommended to minimize attrition bias, in particular when auxiliary variables are available [31, 32]. Besides our variables of interest and covariates, we used the following variables as predictors for imputation because they have been related to pregnancy intention and/or child behavior problems and contain only a small amount of missing data: four digit zip code at birth, parity, maternal pre-pregnancy body mass index, folic acid intake, number of sexual partners in the year prior to pregnancy; and child ethnicity, birth weight and Apgar score after 5 min. These variables were all self-reported, except for birthweight and Apgar score.

Though our method shares some similarities with mediation analyses, it does not estimate the controlled or natural direct or indirect effects, which target the effect of changing the ‘exposure’ while fixing the value of the mediator [33]. Changing the ‘exposure’ (unintended pregnancy) would be very complicated, if not impossible, because the counterfactual of an unintended pregnancy is either an intended pregnancy or no pregnancy at all. In practice, it would only be possible to prevent unintended pregnancies. Hence, the prevention of unintended pregnancies (no pregnancy) would result in an absence of the outcome (childhood behavior). Therefore, we examined whether hypothetically eliminating postnatal depression and increasing social support may improve internalizing and externalizing problems in children of unintended pregnancies and thereby have the potential to reduce the differences in behavioral outcomes between children of intended and unintended pregnancies. This modelling approach is called G-estimation [13, 34].

As a first step we estimated the observed difference in problem behavior, and the association between postnatal depression, social support and problem behavior based on the observed data. Second, we modelled a situation in which all participants were set to ‘no postnatal depression’ and ‘high social support’. Third, we used the models of the second step to estimate the difference in problem behavior after postnatal depression was hypothetically eliminated and social support was hypothetically increased. The epidemiological design requires three causal assumptions: exchangeability, positivity and consistency. Exchangeability means no residual confounding or selection bias in the relationship of postnatal depression and social support with child behavior. We therefore adjusted for the earlier mentioned covariates [26–29]. Positivity indicates enough variation in postnatal depression and social support by levels of the covariates, which is likely satisfied given the sample size of the study population and there are no known structural reasons why positivity would be violated. Consistency requires well defined interventions, which might be violated because postnatal depression and lack of social support can be intervened on in different ways possibly resulting in different causal effects. This should be kept in mind when interpreting the results.

First, differences in internalizing and externalizing problems between pregnancy intention groups were estimated using linear regression models adjusted for child age and sex. Second, the associations of (1) postnatal depression, and (2) social support with internalizing and externalizing problems were estimated using linear regression analyses adjusted for the earlier mentioned covariates [26–29]. This model was subsequently used to obtain the estimated child behavioral outcomes if all study participants were set to ‘no postnatal depression’ or ‘high social support’. By comparing child behavior before and after adjusting postnatal depression and social support, the reduction in the differences in child behavior was estimated [13]. All analyses were run for internalizing and externalizing problems at ages 1.5, 3, 6, 9 and 13 years separately. Bootstrapping with 1000 iterations was used to calculate the 95% confidence intervals. Several sensitivity analyses were performed. Firstly, we used child self-reported internalizing and externalizing problems from the BPM at age 9 years and the YSR at age 13 years to determine whether reporting bias may have influenced our results. Secondly, in women who participated in the study with multiple pregnancies and/or women who gave birth to twins, a random child was excluded (analytical sample *N* = 9621). Thirdly, complete case analyses were performed by excluding dyads with missing data on unintended pregnancy, postnatal depression, social support or all child behavior questionnaires (*N* = 4244 for analyses with postnatal depression, and *N* = 3484 for analyses with social support). Finaly, as posthoc analyses, we repeated the analyses with dichotomized outcomes to assess the reduction in borderline clinical cases if all postnatal depression cases would be eliminated. All analyses were conducted in IBM SPSS version 28 and R statistical software version 4.2.1.

## Results

The mothers were on average 29.9 (SD = 5.4) years old and 72.9% of them had a planned pregnancy, 14.8% had a wanted unplanned pregnancy, 10.8% had an unplanned pregnancy with initially ambivalent feelings and 1.5% had an unplanned pregnancy with prolonged ambivalent feelings. Most mothers were married (49.6%) or cohabiting (25.8%), had a low (26.6%) or mid-low (30.7%) educational level and around half of them (49.8%) had a migration background. Table 1 shows the characteristics of the mothers per pregnancy intention group, and figure S1 shows the correlations between all variables of interest. The proportion of postnatal depression differed significantly between the pregnancy intention groups (6.2% for planned, 9.4% for unplanned and wanted, 15.1% for unplanned with initially ambivalent feelings, and 25.0% for unplanned with prolonged ambivalent feelings). In addition, the average scores of social support differed significantly between the pregnancy intention groups (33.9 (SD = 6.6) for planned, 33.1 (SD = 7.0) for unplanned and wanted, 32.2 (SD = 7.6) for unplanned with initially ambivalent feelings, and 30.9 (SD = 6.5) for unplanned with prolonged ambivalent feelings).

**Table 1:** General characteristics of the pregnant mothers

	Planned pregnancy (*N* = 5729)	Unplanned and wanted from the start (*N* = 1163)	Unplanned and initially ambivalent feelings (*N* = 850)	Unplanned and prolonged ambivalent feelings (*N* = 114)	Missing data on pregnancy intention (*N* = 1765)^3^	*P*-value
**Age mother**						< 0.01^4^
Mean (SD)	30.79 (4.76)	27.95 (5.82)	27.22 (5.94)	28.95 (6.11)	29.73 (5.87)	
N	5729	1163	850	114	1765	
**Marital Status**						< 0.01^5^
Married	3116 (55.6%)	368 (32.8%)	262 (31.9%)	43 (38.4%)	378 (51.4%)	
Cohabiting	2130 (38.0%)	418 (37.3%)	227 (27.6%)	22 (19.6%)	207 (28.2%)	
Single	358 (6.4%)	335 (29.9%)	332 (40.4%)	47 (42.0%)	150 (20.4%)	
N	5604	1121	821	112	735	
**Migration background**						< 0.01^5^
No	3343 (58.8%)	423 (36.9%)	235 (28.0%)	30 (26.5%)	450 (39.4%)	
Yes	2338 (41.2%)	722 (63.1%)	603 (72.0%)	83 (73.5%)	693 (60.6%)	
N	5681	1145	838	113	1143	
**Educational level mother** ^**1**^						< 0.01^5^
Low	1160 (20.9%)	397 (35.7%)	359 (44.3%)	46 (42.2%)	268 (33.4%)	
Mid-Low	1601 (28.8%)	408 (36.7%)	297 (36.6%)	30 (27.5%)	238 (29.7%)	
Mid-High	1192 (21.5%)	181 (16.3%)	94 (11.6%)	14 (12.8%)	149 (18.6%)	
High	1603 (28.9%)	126 (11.3%)	61 (7.5%)	19 (17.4%)	147 (18.3%)	
N	5556	1112	811	109	802	
**Household income** ^**2**^						< 0.01^5^
Low	520 (11.9%)	269 (33.1%)	263 (46.8%)	48 (52.7%)	258 (35.4%)	
Medium	710 (16.2%)	189 (23.2%)	143 (25.4%)	22 (24.2%)	151 (20.7%)	
High	3143 (71.9%)	355 (43.7%)	156 (27.8%)	21 (23.1%)	320 (43.9%)	
N	4373	813	562	91	729	
**Parity**						< 0.01^5^
0	3197 (57.1%)	695 (60.6%)	463 (56.1%)	45 (40.5%)	678 (46.8%)	
1	1825 (32.6%)	307 (26.8%)	180 (21.8%)	34 (30.6%)	451 (31.1%)	
2	467 (8.3%)	113 (9.9%)	138 (16.7%)	21 (18.9%)	230 (15.9%)	
≥3	107 (1.9%)	31 (2.7%)	45 (5.4%)	11 (9.9%)	90 (6.2%)	
N	5596	1146	826	111	1449	
**Postnatal depression**						< 0.01^5^
No	3153 (93.8%)	465 (90.6%)	270 (84.9%)	39 (75.0%)	482 (86.8%)	
Yes	208 (6.2%)	48 (9.4%)	48 (15.1%)	13 (25.0%)	73 (13.2%)	
N	3361	513	318	52	555	
**Social support**						< 0.01^4^
Mean (SD)	33.94 (6.55)	33.12 (6.99)	32.23 (7.61)	30.94 (6.53)	32.38 (7.60)	
N	2889	396	254	47	488	

^1^ Highest attained educational level, categorized as: Low (primary school; lower vocational training; intermediate general school; 3 years general secondary school), which typically corresponds to ≤ 12 years of education; Mid-low (> 3 years general secondary school; intermediate vocational training; 1st year higher vocational training), in general corresponding with 13–15 years of education; Mid‐high (higher vocational training; Bachelor’s degree), typically matching with 16 or 17 years of education; And High (higher academic education; PhD), usually indicating 18 years of education or more

^2^ Categorized as: Less than €1200/month (social security level); Between €1200 and €2000/month; and More than €2000/month (modal income)

^3^ Pregnancy intention data was missing for *n* = 1765 participants

^4^ Kruskal-Wallis rank sum test

^5^ Pearson’s Chi-squared test

**Fig. 1:**
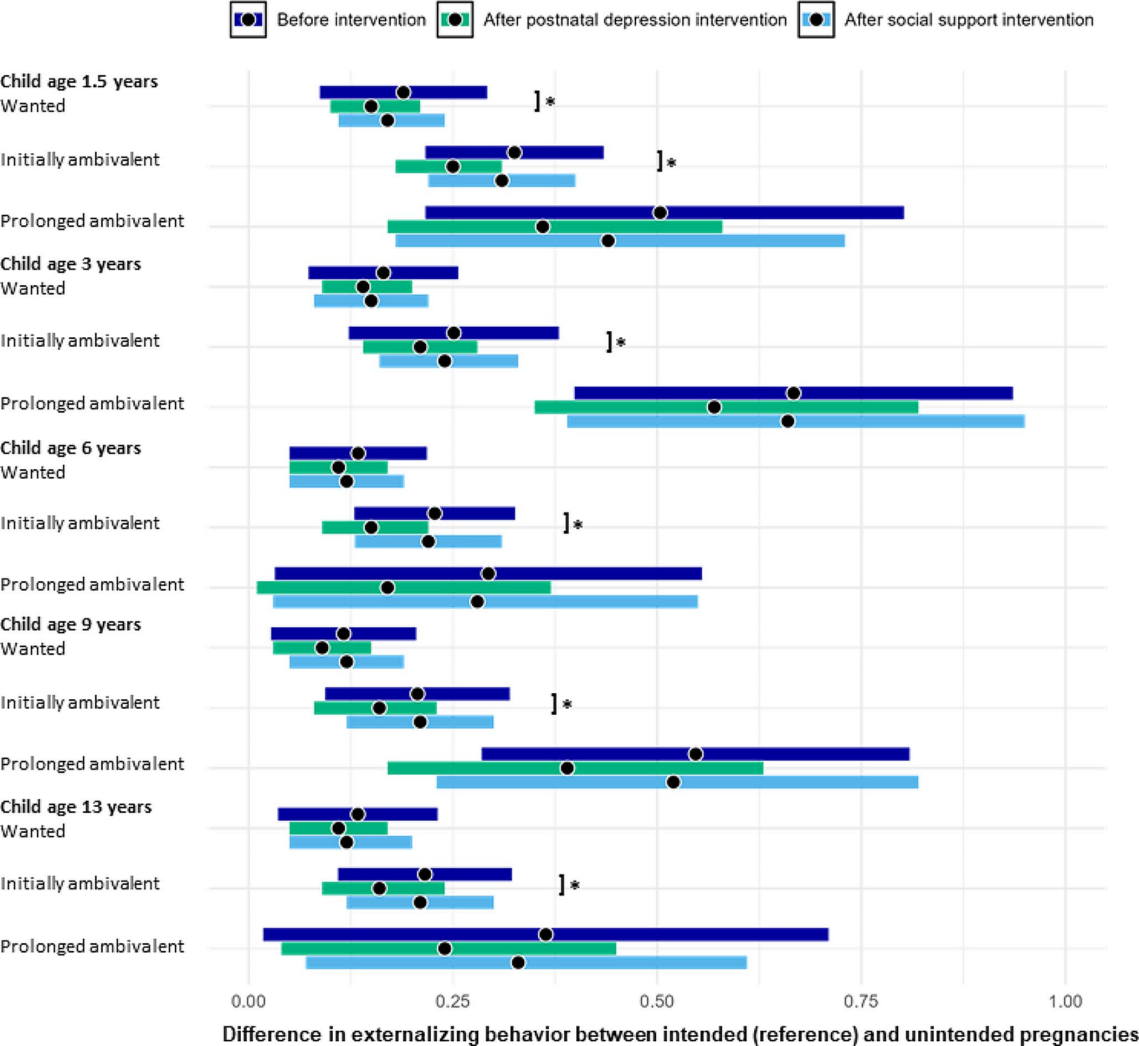
Forest plot of the differences in internalizing problem behavior (dark blue), after hypothetically reducing postnatal depression (green), and after hypothetically increasing social support (light blue) between children from planned pregnancies (reference) and children from unplanned and wanted pregnancies, unplanned pregnancies with initially ambivalent feelings and unplanned pregnancies with prolonged ambivalent feelings ages 1.5, 3, 6, 9 and 13 years. The beta-coefficients are indicated with a black dot, and the 95% confidence intervals are indicated by the width of the error bars

**Fig. 2:**
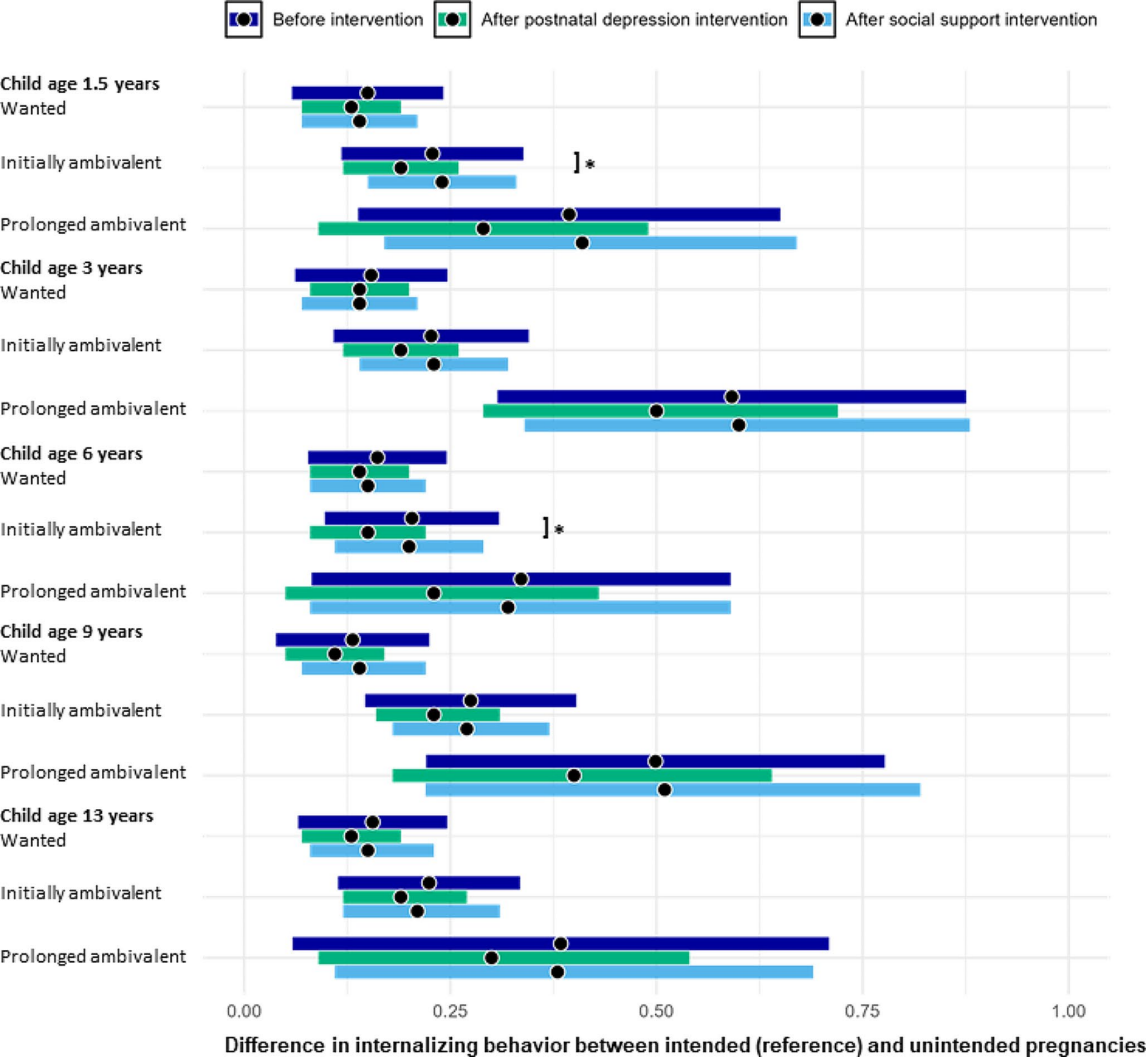
Forest plot of the differences in externalizing problem behavior before the hypothetical intervention (dark blue), after hypothetically reducing postnatal depression (green), and after hypothetically increasing social support (light blue) between children from planned pregnancies (reference) and children from unplanned and wanted pregnancies, unplanned pregnancies with initially ambivalent feelings and unplanned pregnancies with prolonged ambivalent feelings ages 1.5, 3, 6, 9 and 13 years. The beta-coefficients are indicated with a black dot, and the 95% confidence intervals are indicated by the width of the error bars

At all ages, children from unplanned pregnancies had significantly more internalizing and externalizing problems as compared to children from planned pregnancies (Figs. 1 and 2; Tables 2 and 3). The difference was largest for children from unplanned pregnancies with prolonged ambivalent feelings and smallest for children from wanted unplanned pregnancies. For example, at age 1.5 years, children from wanted unplanned pregnancies had β = 0.19 (95% CI = 0.13; 0.25) more internalizing and β = 0.15 (95% CI = 0.09; 0.21) more externalizing problems; children from an unplanned pregnancy with initially ambivalent feelings had β = 0.33 (95% CI = 0.25; 0.40) more internalizing and β = 0.23 (95% CI = 0.16; 0.30) more externalizing problems; and children from an unplanned pregnancy with prolonged ambivalent feelings had β = 0.50 (95% CI = 0.30; 0.72) more internalizing and β = 0.39 (95% CI = 0.22; 0.57) more externalizing problems as compared to children from planned pregnancies.

**Table 2:** Changes in behavior differences by hypothetically eliminating postnatal depression

	Internalizing behavior			Externalizing behavior		
	Before	After eliminating postnatal depression	Reduction	Before	After eliminating postnatal depression	Reduction
**Age 1.5 years**						
Unplanned and wanted	0.19 (0.13; 0.25)	0.15 (0.10; 0.21)	0.03 (7.5^e − 4^; 0.07)*	0.15 (0.09–0.21)	0.13 (0.07; 0.19)	0.02 (-4.6^e − 3^; 0.05)
Unplanned and initially ambivalent	0.33 (0.25; 0.40)	0.25 (0.18; 0.31)	0.08 (0.03; 0.13)*	0.23 (0.16–0.30)	0.19 (0.12; 0.26)	0.04 (2.2^e − 3^; 0.08)*
Unplanned and prolonged ambivalent	0.50 (0.30; 0.72)	0.36 (0.17; 0.58)	0.14 (-0.02; 0.32)	0.39 (0.22–0.57)	0.29 (0.09; 0.49)	0.11 (-0.03; 0.25)
**Age 3 years**						
Unplanned and wanted	0.16 (0.11; 0.22)	0.14 (0.09; 0.20)	0.02 (-0.01; 0.05)	0.15 (0.10–0.21)	0.14 (0.08; 0.20)	0.02 (-0.01; 0.05
Unplanned and initially ambivalent	0.25 (0.18; 0.32)	0.21 (0.14; 0.28)	0.04 (1.2^e − 3^; 0.09)*	0.23 (0.16–0.30)	0.19 (0.12; 0.26)	0.04 (-3.1^e − 3^; 0.08)
Unplanned and prolonged ambivalent	0.67 (0.46; 0.89)	0.57 (0.35; 0.82)	0.10 (-0.07; 0.27)	0.59 (0.40–0.78)	0.50 (0.39; 0.72)	0.09 (-0.06; 0.25)
**Age 6 years**						
Unplanned and wanted	0.13 (0.08; 0.19)	0.11 (0.05; 0.17)	0.02 (-0.01; 0.06)	0.16 (0.10; 0.22)	0.14 (0.08; 0.20)	0.02 (-0.01; 0.05)
Unplanned and initially ambivalent	0.23 (0.16; 0.30)	0.15 (0.09; 0.22)	0.07 (0.03; 0.12)*	0.20 (0.13; 0.27)	0.15 (0.08; 0.20)	0.06 (0.02; 0.10)*
Unplanned and prolonged ambivalent	0.29 (0.10; 0.49)	0.17 (0.01; 0.37)	0.12 (-0.03; 0.29)	0.34 (0.15; 0.53)	0.23 (0.05; 0.43)	0.10 (-0.05; 0.10)
**Age 9 years**						
Unplanned and wanted	0.12 (0.06; 0.18)	0.09 (0.03; 0.15)	0.03 (-4.9^e − 3^; 0.06)	0.13 (0.07; 0.19)	0.11 (0.05; 0.17)	0.02 (-0.01; 0.05)
Unplanned and initially ambivalent	0.21 (0.14; 0.28)	0.16 (0.08; 0.23)	0.05 (0.01; 0.09)*	0.27 (0.20; 0.35)	0.23 (0.16; 0.31)	0.04 (-4.2^e − 3^; 0.09)
Unplanned and prolonged ambivalent	0.55 (0.34; 0.76)	0.39 (0.17; 0.63)	0.16 (-0.01; 0.33)	0.50 (0.30; 0.72)	0.40 (0.18; 0.64)	0.10 (-0.06; 0.27)
**Age 13 years**						
Unplanned and wanted	0.13 (0.08; 0.19)	0.11 (0.05; 0.17)	0.02 (-0.01; 0.05)	0.16 (0.10; 0.22)	0.13 (0.07; 0.19)	0.02 (-0.01; 0.06)
Unplanned and initially ambivalent	0.22 (0.15; 0.29)	0.16 (0.09; 0.24)	0.05 (0.01; 0.10)*	0.22 (0.15; 0.30)	0.19 (0.12; 0.27)	0.03 (-0.01; 0.07)
Unplanned and prolonged ambivalent	0.36 (0.18; 0.56)	0.24 (0.04; 0.45)	0.13 (-0.02; 0.29)	0.38 (0.19; 0.60)	0.30 (0.09; 0.54)	0.08 (-0.08; 0.25)

* Significant reduction. The 95% confidence intervals were calculated by bootstrapping with 1000 iterations

Before: The absolute difference in internalizing and externalizing problem behaviour (beta-coefficients) between the intention groups with planned pregnancy as reference and 95% confidence interval in between brackets

After eliminating postnatal depression: The absolute difference in internalizing and externalizing problem behaviour (beta-coefficients) between the intention groups with planned pregnancy as reference and 95% confidence interval in between brackets after setting the study population to ‘no postnatal depression’

Reduction: The absolute change of the beta-coefficients without and with intervention in internalizing and externalizing problem behaviour between the intention groups with planned pregnancy as reference and 95% confidence interval in between brackets

**Table 3:** Changes in behavior differences by hypothetically increasing social support

	Internalizing behavior			Externalizing behavior		
	Before	After increasing social support	Reduction	Before	After increasing social support	Reduction
**Age 1.5 years**						
Unplanned and wanted	0.19 (0.13; 0.25)	0.17 (0.11; 0.24)	0.01 (-0.03; 0.06)	0.15 (0.09–0.21)	0.14 (0.07; 0.21)	0.01 (-0.04; 0.06)
Unplanned and initially ambivalent	0.33 (0.25; 0.40)	0.31 (0.22; 0.40)	0.01 (-0.05; 0.08)	0.23 (0.16–0.30)	0.24 (0.15; 0.33)	-0.01 (-0.07; 0.05)
Unplanned and prolonged ambivalent	0.50 (0.30; 0.72)	0.44 (0.18; 0.73)	0.06 (-0.15; 0.28)	0.39 (0.22–0.57)	0.41 (0.17; 0.67)	-0.02 (-0.21; 0.17)
**Age 3 years**						
Unplanned and wanted	0.16 (0.11; 0.22)	0.15 (0.08; 0.22)	0.01 (-0.03; 0.06)	0.15 (0.10–0.21)	0.14 (0.07; 0.21)	0.01 (-0.03; 0.06)
Unplanned and initially ambivalent	0.25 (0.18; 0.32)	0.24 (0.16; 0.33)	0.01 (-0.05; 0.07)	0.23 (0.16–0.30)	0.23 (0.14; 0.32)	0.00 (-0.06; 0.06)
Unplanned and prolonged ambivalent	0.67 (0.46; 0.89)	0.66 (0.39; 0.95)	0.01 (-0.21; 0.24)	0.59 (0.40–0.78)	0.60 (0.34; 0.88)	-0.01 (0.19; 0.22)
**Age 6 years**						
Unplanned and wanted	0.13 (0.08; 0.19)	0.12 (0.05; 0.19)	0.01 (-0.03; 0.06)	0.16 (0.10; 0.22)	0.15 (0.08; 0.22)	0.01 (-0.04; 0.06)
Unplanned and initially ambivalent	0.23 (0.16; 0.30)	0.22 (0.13; 0.31)	0.01 (-0.05; 0.08)	0.20 (0.13; 0.27)	0.20 (0.11; 0.29)	0.00 (-0.06; 0.07)
Unplanned and prolonged ambivalent	0.29 (0.10; 0.49)	0.28 (0.03; 0.55)	0.01 (-0.19; 0.22)	0.34 (0.15; 0.53)	0.32 (0.08; 0.59)	0.01 (-0.19; 0.21)
**Age 9 years**						
Unplanned and wanted	0.12 (0.06; 0.18)	0.12 (0.05; 0.19)	0.00 (-0.05; 0.05)	0.13 (0.07; 0.19)	0.14 (0.07; 0.22)	-0.01 (-0.06; 0.04)
Unplanned and initially ambivalent	0.21 (0.14; 0.28)	0.21 (0.12; 0.30)	0.00 (-0.07; 0.06)	0.27 (0.20; 0.35)	0.27 (0.18; 0.37)	0.00 (-0.06; 0.07)
Unplanned and prolonged ambivalent	0.55 (0.34; 0.76)	0.52 (0.23; 0.82)	0.03 (-0.19; 0.25)	0.50 (0.30; 0.72)	0.51 (0.22; 0.82)	-0.01 (-0.24; 0.21)
**Age 13 years**						
Unplanned and wanted	0.13 (0.08; 0.19)	0.12 (0.05; 0.20)	0.01 (-0.04; 0.06)	0.16 (0.10; 0.22)	0.15 (0.08; 0.23)	0.00 (-0.05; 0.05)
Unplanned and initially ambivalent	0.22 (0.15; 0.29)	0.21 (0.12; 0.30)	0.01 (-0.05; 0.07)	0.22 (0.15; 0.30)	0.21 (0.12; 0.31)	0.01 (-0.05; 0.08)
Unplanned and prolonged ambivalent	0.36 (0.18; 0.56)	0.33 (0.07; 0.61)	0.03 (-0.17; 0.24)	0.38 (0.19; 0.60)	0.38 (0.11; 0.69)	0.00 (-0.22; 0.22)

Before: The absolute difference in internalizing and externalizing problem behaviour (beta-coefficients) between the intention groups with planned pregnancy as reference and 95% confidence interval in between brackets

After increasing social support: The absolute difference in internalizing and externalizing problem behaviour (beta-coefficients) between the intention groups with planned pregnancy as reference and 95% confidence interval in between brackets after setting the study population to ‘sufficient social support’

Reduction: The absolute change of the beta-coefficients without and with intervention in internalizing and externalizing problem behaviour between the intention groups with planned pregnancy as reference and 95% confidence interval in between brackets

The 95% confidence intervals were calculated by bootstrapping with 1000 iterations

Hypothetically eliminatingpostnatal depression reduced the differences in offspring internalizing and externalizing problems to some extent, with largest reductions seen for unplanned pregnancies with prolonged ambivalent feelings (Table 2). For example, at age 1.5 years, the reduction of planned vs. unplanned and wanted pregnancies was β = 0.03 (95% CI = 0.00; 0.07) for internalizing and β = 0.02 (95% CI=-4.6e-3; 0.05) for externalizing problems. The reduction of planned vs. unplanned pregnancies with initially ambivalent feelings was β = 0.08 (95% CI = 0.03; 0.13) for internalizing and β = 0.04 (95% CI = 2.2e-3; 0.08) for externalizing problems. The reduction of planned vs. unplanned pregnancies with prolonged ambivalent feelings was β = 0.14 (95% CI=-0.02; 0.32) for internalizing and β = 0.04 (95% CI=-0.03; 0.25) for externalizing problems. Reductions were quite comparable across ages. Hypothetically increasing social support did not result in significant changes in differences in internalizing problems and externalizing problems between the different pregnancy intention groups at all ages (Table 3).

Sensitivity analyses using child self-reported internalizing and externalizing problems (*n* = 9260) and excluding a random child in case women participated in the study with multiple children (*n* = 8892) both showed similar results (Supplemental Tables 2–5). Complete case analyses excluding dyads with missing data on unintended pregnancy, postnatal depression, social support or child behavior questionnaires (leaving *n* = 4034 for postnatal depression and *n* = 3484 for social support) showed a smaller difference in internalizing and externalizing problems between planned and unplanned pregnancies. Hypothetically eliminatingpostnatal depression showed smaller and non-significant reductions in the differences, while hypothetically increasing social support showed non-significant increases in the differences with very wide confidence intervals (Supplemental Tables 6 and 7).

Post-hoc analyses with borderline clinical cases as outcome showed large differences in proportions of borderline clinical cases across the pregnancy groups. For example, at age 1.5 years, children from wanted unplanned pregnancies had 4.8% (95% CI = 2.9%; 6.7%) more internalizing and 4.8% (95% CI = 2.6%; 7.0%) more externalizing borderline clinical cases; children from an unplanned pregnancy with initially ambivalent feelings had 8.4% (95% CI = 6.1%; 10.8%) more internalizing and 8.3% (95% CI = 5.8%; 11.0%) more externalizing borderline clinical cases; and children from an unplanned pregnancy with prolonged ambivalent feelings had 12.1% (95% CI = 5.6%; 18.9%) more internalizing and 12.5% (95% CI = 5.7%; 19.9%) more externalizing borderline clinical cases as compared to children from planned pregnancies. Hypothetically eliminating postnatal depression would reduce these differences by 0.6% (95% CI=-1.7%; 0.5%) to 4.9% (95% CI=-11.0%; 0.9%) in internalizing, and − 0.5% (95% CI=-1.5%; 0.4%) to -3.7% (95% CI=-8.8%; 0.9%) in externalizing borderline clinical cases (Supplemental Table 8).

## Discussion

The results of this study showed that children from unintended pregnancies had more internalizing and externalizing problems as compared to children from intended pregnancies across the childhood years into early adolescence. Problem behavior in the children was higher when the mother had more ambivalent feelings towards the unplanned pregnancy. Hypothetically eliminating postnatal depression reduced the differences between intended and unintended pregnancies by 0.02 (95% CI= -0.01; 0.05) to 0.16 standard deviation (95% CI=-0.01; 0.33) for internalizing problems; and 0.02 (95% CI=-0.01;0.05) to 0.11 standard deviation (95% CI=-0.03; 0.25) for externalizing problems. Surprisingly, the results suggested that hypothetically increasing social support would not result in any reductions in internalizing and externalizing problems in the offspring.

Previous studies were in line with our findings and demonstrated more internalizing and externalizing problems in children from women with unplanned or unwanted pregnancy as compared to planned pregnancies [4, 5, 35, 36]. Only few studies focused on the potential pathways behind this association. In 14-year-old Australian children, the association between (un)planned pregnancy and internalizing and externalizing problems was reduced by 50% after adjustment for potential confounders (mother’s age, mother’s marital status, mother’s anxiety, depression and smoking at first clinic visit). Additional adjustment for mother’s attitudes towards caring for a baby at 6 months did not change the results [5]. In children aged 5 and 7 years from the United Kingdom, child behavior was measured using the Strengths and Difficulties Questionnaire. Children from unplanned and mistimed pregnancies had more difficulties than children from planned pregnancies. Again, the association was reduced by 50% after adjustment for pregnancy and postnatal factors, such as gestational age, alcohol drinking, smoking, breastfeeding, maternal mental health, attachment and parenting [4]. These studies suggest that predisposing or coinciding factors may result in behavioral problems in children from unintended pregnancies. The statistical design of these studies hamper causal interpretation, because of violating the exchangebility assumption due to adjustment for potential colliders (postnatal factors) [33, 37]. We therefore applied G-estimation, that has the advantage to provide estimates under less restrictive identification conditions. Our results provided more solid evidence that reducing postnatal depression symptoms may reduce the association between (un)planned pregnancy and internalizing and externalizing problem behavior. The inequality would be reduced by 14–36% in an ideal setting in which all postnatal depression would be *fully* eliminated. This suggests that a more comprehensive intervention focusing on, amongst others, maternal mental health, substance use during pregnancy, child-parent attachment and parenting is needed in order to reduce inequalities in child behavior.

Our findings suggest that preventing postnatal depression may be promising to reduce differences in behavior problems between children from intended versus unintended pregnancies, in particular when ambivalent feelings towards the pregnancy are present. Some studies have shown that mothers who had an unintended pregnancy more often suffered from postnatal depression [38, 39]. A recent study also showed that ambivalent feelings towards the pregnancy were associated with psychological distress in mothers within the first year after childbirth [40]. However, one of the strongest predictors for postnatal depression is prenatal depression [41]. Therefore, it is very important to screen and treat depressive symptoms already during pregnancy, particularly among unplanned pregnant women who experience ambivalent feelings. This may help to reduce disparities in child behavior later in life.

We did not find evidence that high social support reduces differences in behavior problems between children from intended versus unintended pregnancies. This is surprising, because many studies provided evidence for an association between lack of social support and decreased maternal mental health [41, 42]. In our study, postnatal depression and social support were slightly negatively correlated (*r*=-0.21), suggesting that receiving more social support may be associated with better maternal mental health. Only one recent study focused on the association between perceived social support of mothers and child behavior and found that social support during pregnancy was associated with less mental and behavioural disorders in Finnisch children from birth to the age of 10 [43]. In our study, however, social support was very weakly correlated with both unintended pregnancy as well as with problem behavior in children (Figure S1). Other studies suggested that support of the partner is important for reducing postnatal depressive symptoms and quality of bonding to the child [44, 45]. Since the Social Support List 12 – Interactions (SSL12-I) was originally developed to measure social support in elderly, it may be less applicable in our sample [24, 25]. Items regarding support of the partner, baby sitting or help with taking care of the child were not included in the SSL12-I.

Strengths of this study were the large sample size, the novel epidemiological design of the study and the longitudinal data which allowed us to study both short- and long-term differences in behavior problems between children from intended versus unintended pregnancies. Another strength of this study is that unintended pregnancy was measured using two questions; whether the pregnancy was planned; and how the women felt about the unplanned pregnancy. Previous studies were often limited by the use of either planned versus unplanned or wanted versus unwanted pregnancy [5], while it is known that an unintended pregnancy is more complex than just unplanned or just unwanted [46].

Also, some limitations should be considered. The differences in behavior problems between intended vs. unintended pregnancies were smaller in the complete cases analyses than in the analyses of the full sample. This may indicate the presence of differential missing data by (un)intended pregnancies. Multiple imputation was used to ensure that all participants were included in the analysis to avoid attrition bias. We included all variables from the analyses, as well as many auxiliary variables in the imputation to minimize bias related to differential missingness [31, 32]. In order to satisfy the exchangeability assumption, we adjusted for many covariates, including maternal and paternal lifetime psychopathology as a proxy for genetic vulnerability. However, unmeasured confounding of the intervention–outcome model may have led to an overestimation of the change in problem behavior differences between intended and unintended pregnancies. This means that we measured the maximum impact these interventions could have in reducing the difference in child behavior in the main analysis. Finally, the complete case analyses showed less significant findings for the hypothetical intervention on postnatal depression than the analyses on the full sample. This may be explained by both the smaller sample size, and by the smaller difference of behavior problems between intended vs. unintended pregnancies.

In conclusion, our study showed that children from unintended pregnancies may encounter more problem behavior than children from intended pregnancies. This difference was observed as early as 1.5 years, and continued to be observed for the next 12 years. Hypothetically eliminating postnatal depression may, to some extent, reduce inequalities in problem behavior related to unintended pregnancy and in this way combat intergenerational transmission of psychopathology. Therefore, it is important to identify and treat mental health problems of unplanned pregnant women, especially in those with ambivalent feelings, since this may reduce the development of child problem behavior from early childhood to adolescence.

## Electronic supplementary material

Below is the link to the electronic supplementary material.

Supplementary Material 1

## Data Availability

Data from this study are available upon reasonable request to the director of the Generation R Study (generationr@erasmusmc.nl), subject to local, national and European rules and regulations.
