# Supplementary material for: Reducing behavior problems in children born after an unintended pregnancy: the generation R study
Source: Soc Psychiatry Psychiatr Epidemiol. 2024 May 31;59(12):2247–58. doi: 10.1007/s00127-024-02693-3 (PMC11522198; doi:10.1007/s00127-024-02693-3)
Supplement: Supplementary file 1 — Supplementary Material 1 [file 127_2024_2693_MOESM1_ESM.pdf]

Supplemental material

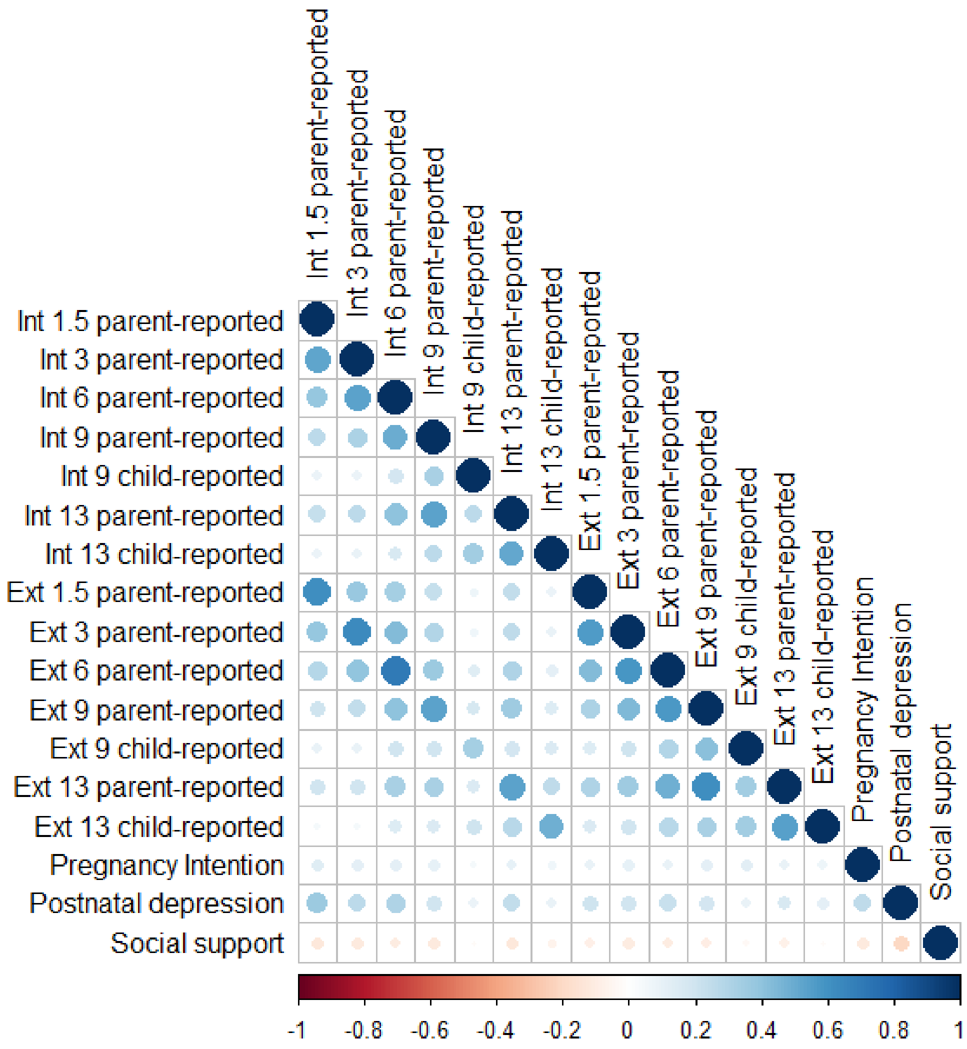

Figure S1: Correlation plot of all variables of interest of the non-imputed data.
Int: Internalizing behavior, Ext: Externalizing behavior.

Table S1: Cronbach's alpha for internalizing and externalizing behavior measured by the Child Behavior Checklist at all ages, the Brief Problem Monitor (self-reported) at age 9 years, and the Youth Self-Report (self-reported) at age 13 years.

	Cronbach's alpha for internalizing behavior	Cronbach's alpha for externalizing behavior
**1.5 years**	0.78	0.88
**3 years**	0.80	0.88
**6 years**	0.86	0.90
**9 years**	0.84	0.87
**9 years self-reported**	0.72	0.63
**13 years**	0.87	0.88
**13 years self-reported**	0.88	0.82

Table S2: Sensitivity analyses with changes in self-reported behavior differences by hypothetically eliminating postnatal depression.

	Internalizing behavior			Externalizing behavior		
	**Before**	**After eliminating postnatal depression**	**Reduction**	**Before**	**After eliminating postnatal depression**	**Reduction**
**Age 9 years**						
Unplanned and wanted	0.05 (2.6^{e-3}; 0.11)	0.03 (-0.03; 0.09)	0.02 (-0.01; 0.05)	0.13 (0.07; 0.19)	0.11 (0.05; 0.17)	0.02 (-0.01; 0.06)
Unplanned and initially ambivalent	0.20 (0.13; 0.26)	0.16 (0.09; 0.24)	0.04 (-2.0^{e-3}; 0.07)	0.32 (0.25; 0.39)	0.29 (0.22; 0.37)	0.02 (-0.02; 0.06)
Unplanned and prolonged ambivalent	0.13 (-0.04; 0.31)	0.04 (-0.15; 0.25)	0.09 (-0.05; 0.23)	0.33 (0.15; 0.52)	0.29 (0.07; 0.52)	0.04 (-0.10; 0.19)
**Age 13 years**						
Unplanned and wanted	0.06 (0.01; 0.12)	0.05 (4.2^{e-3}; 0.11)	0.01 (-0.03; 0.03)	0.18 (0.12; 0.24)	0.16 (0.10; 0.23)	0.02 (-0.01; 0.06)
Unplanned and initially ambivalent	0.21 (0.15; 0.28)	0.19 (0.11; 0.26)	0.03 (-0.01; 0.07)	0.20 (0.14; 0.27)	0.19 (0.11; 0.27)	0.02 (-0.02; 0.06)
Unplanned and prolonged ambivalent	0.21 (0.03; 0.40)	0.17 (-0.04; 0.39)	0.04 (-0.10; 0.18)	0.17 (-1.9^{e-3}; 0.35)	0.14 (-0.06; 0.35)	0.03 (-0.10; 0.17)

The 95% confidence intervals were calculated by bootstrapping with 1000 iterations.

Before: The absolute difference in internalizing and externalizing problem behaviour (beta-coefficients) between the intention groups with planned pregnancy as reference and 95% confidence interval in between brackets.

After eliminating postnatal depression: The absolute difference in internalizing and externalizing problem behaviour (beta-coefficients) between the intention groups with planned pregnancy as reference and 95% confidence interval in between brackets after setting the study population to ‘no postnatal depression’.

Reduction: The absolute change of the beta-coefficients without and with intervention in internalizing and externalizing problem behaviour between the intention groups with planned pregnancy as reference and 95% confidence interval in between brackets.

Table S3: Sensitivity analyses with changes in self-reported behavior differences by hypothetically increasing social support.

	Internalizing behavior			Externalizing behavior		
	**Before**	**After increasing social support**	**Reduction**	**Before**	**After increasing social support**	**Reduction**
**Age 9 years**						
Unplanned and wanted	0.05 (2.6^{e-3}; 0.11)	0.06 (-0.01; 0.14)	-0.01 (-0.06; 0.04)	0.13 (0.07; 0.19)	0.12 (0.05; 0.20)	0.01 (-0.04; 0.05)
Unplanned and initially ambivalent	0.20 (0.13; 0.26)	0.20 (0.11; 0.29)	-0.01 (-0.07; 0.06)	0.32 (0.25; 0.39)	0.32 (0.23; 0.42)	0.00 (-0.07; 0.06)
Unplanned and prolonged ambivalent	0.13 (-0.04; 0.31)	0.17 (-0.08; 0.44)	-0.04 (-0.23; 0.15)	0.33 (0.15; 0.52)	0.36 (0.09; 0.65)	-0.03 (-0.24; 0.17)
**Age 13 years**						
Unplanned and wanted	0.06 (0.01; 0.12)	0.06 (4.2^{e-3}; 0.13)	0.00 (-0.05; 0.04)	0.18 (0.12; 0.24)	0.17 (0.10; 0.25)	0.00 (-0.04; 0.05)
Unplanned and initially ambivalent	0.21 (0.15; 0.28)	0.21 (0.12; 0.30)	0.01 (-0.05; 0.07)	0.20 (0.14; 0.27)	0.20 (0.11; 0.29)	0.01 (-0.06; 0.07)
Unplanned and prolonged ambivalent	0.21 (0.03; 0.40)	0.25 (-0.01; 0.53)	-0.04 (-0.24; 0.16)	0.17 (-1.9^{e-3}; 0.35)	0.17 (0.08; 0.43)	0.00 (-0.18; 0.19)

The 95% confidence intervals were calculated by bootstrapping with 1000 iterations.

Before: The absolute difference in internalizing and externalizing problem behaviour (beta-coefficients) between the intention groups with planned pregnancy as reference and 95% confidence interval in between brackets.

After increasing social support: The absolute difference in internalizing and externalizing problem behaviour (beta-coefficients) between the intention groups with planned pregnancy as reference and 95% confidence interval in between brackets after setting the study population to 'sufficient social support'.

Reduction: The absolute change of the beta-coefficients without and with intervention in internalizing and externalizing problem behaviour between the intention groups with planned pregnancy as reference and 95% confidence interval in between brackets.

The 95% confidence intervals were calculated by bootstrapping with 1000 iterations.

Table S4: Changes in behavior differences by hypothetically eliminating postnatal depression excluding a random child in case of siblings.**

	**Internalizing behavior**			**Externalizing behavior**		
	**Before**	**After eliminating postnatal depression**	**Reduction**	**Before**	**After eliminating postnatal depression**	**Reduction**
**Age 1.5 years**						
Unplanned and wanted	0.19 (0.13; 0.25)	0.15 (0.10; 0.21)	0.03 (-2.2^{e-3}; 0.07)	0.15 (0.09-0.21)	0.13 (0.07; 0.19)	0.02 (-0.01; 0.05)
Unplanned and initially ambivalent	0.32 (0.25; 0.39)	0.24 (0.17; 0.31)	0.08 (0.03; 0.07)*	0.23 (0.16-0.30)	0.19 (0.11; 0.26)	0.04 (8.9^{e-4}; 0.08)*
Unplanned and prolonged ambivalent	0.48 (0.27; 0.71)	0.35 (0.15; 0.58)	0.13 (-0.04; 0.31)	0.38 (0.19-0.56)	0.27 (0.07; 0.48)	0.10 (-0.04; 0.25)
**Age 3 years**						
Unplanned and wanted	0.16 (0.10; 0.22)	0.14 (0.08; 0.20)	0.02 (-0.01; 0.06)	0.15 (0.09-0.21)	0.13 (0.07; 0.19)	0.02 (-0.01; 0.05)
Unplanned and initially ambivalent	0.24 (0.17; 0.31)	0.20 (0.12; 0.27)	0.04 (6.1^{e-4}; 0.09)*	0.22 (0.15-0.29)	0.18 (0.11; 0.26)	0.04 (-0.01; 0.08)
Unplanned and prolonged ambivalent	0.65 (0.43; 0.88)	0.56 (0.32; 0.81)	0.09 (-0.08; 0.28)	0.56 (0.37-0.76)	0.47 (0.26; 0.70)	0.09 (-0.06; 0.25)
**Age 6 years**						
Unplanned and wanted	0.13 (0.07; 0.20)	0.11 (0.05; 0.17)	0.03 (-0.01; 0.06)	0.16 (0.10; 0.22)	0.13 (0.07; 0.20)	0.02 (-0.01; 0.06)
Unplanned and initially ambivalent	0.22 (0.15; 0.30)	0.15 (0.08; 0.22)	0.08 (0.03; 0.13)*	0.20 (0.13; 0.27)	0.14 (0.07; 0.21)	0.06 (0.02; 0.10)*
Unplanned and prolonged ambivalent	0.30 (0.10; 0.50)	0.17 (0.02; 0.37)	0.13 (-0.03; 0.30)	0.34 (0.14; 0.54)	0.23 (0.03; 0.44)	0.11 (-0.05; 0.27)
**Age 9 years**						
Unplanned and wanted	0.12 (0.06; 0.18)	0.10 (0.03; 0.16)	0.03 (-0.01; 0.06)	0.13 (0.07; 0.19)	0.11 (0.05; 0.17)	0.02 (-0.01; 0.06)
Unplanned and initially ambivalent	0.20 (0.13; 0.28)	0.15 (0.08; 0.23)	0.05 (0.01; 0.10)*	0.27 (0.20; 0.35)	0.23 (0.15; 0.31)	0.04 (-2.8^{e-3}; 0.09)
Unplanned and prolonged ambivalent	0.52 (0.30; 0.75)	0.37 (0.14; 0.62)	0.14 (-0.03; 0.33)	0.48 (0.27; 0.71)	0.37 (0.15; 0.62)	0.11 (-0.06; 0.29)
**Age 13 years**						
Unplanned and wanted	0.14 (0.08; 0.20)	0.12 (0.05; 0.18)	0.02 (-0.01; 0.05)	0.16 (0.09; 0.22)	0.13 (0.06; 0.19)	0.03 (-0.01; 0.06)
Unplanned and initially ambivalent	0.21 (0.14; 0.28)	0.16 (0.08; 0.23)	0.05 (0.01; 0.10)*	0.22 (0.15; 0.30)	0.19 (0.11; 0.27)	0.03 (-0.01; 0.08)
Unplanned and prolonged ambivalent	0.33 (0.14; 0.54)	0.21 (0.01; 0.43)	0.13 (-0.03; 0.29)	0.37 (0.17; 0.59)	0.28 (0.06; 0.53)	0.09 (-0.07; 0.27)

* Significant reduction. The 95% confidence intervals were calculated by bootstrapping with 1000 iterations.

** In women who participated in the study with multiple pregnancies or who gave birth to twins, a random child was excluded, leaving N=8892 participants.

Before: The absolute difference in internalizing and externalizing problem behaviour (beta-coefficients) between the intention groups with planned pregnancy as reference and 95% confidence interval in between brackets.

After eliminating postnatal depression: The absolute difference in internalizing and externalizing problem behaviour (beta-coefficients) between the intention groups with planned pregnancy as reference and 95% confidence interval in between brackets after setting the study population to 'no postnatal depression'.

Reduction: The absolute change of the beta-coefficients without and with intervention in internalizing and externalizing problem behaviour between the intention groups with planned pregnancy as reference and 95% confidence interval in between brackets.

Table S5: Changes in behavior differences by hypothetically increasing social support using excluding a random child in case of siblings*.

	Internalizing behavior			Externalizing behavior		
	Before	After increasing social support	Reduction	Before	After increasing social support	Reduction
**Age 1.5 years**						
Unplanned and wanted	0.19 (0.13; 0.25)	0.18 (0.11; 0.25)	0.01 (-0.04; 0.06)	0.15 (0.09-0.21)	0.14 (0.07; 0.22)	0.01 (-0.04; 0.05)
Unplanned and initially ambivalent	0.32 (0.25; 0.39)	0.31 (0.22; 0.40)	0.01 (-0.06; 0.07)	0.23 (0.16-0.30)	0.24 (0.15; 0.33)	-0.02 (-0.08; 0.05)
Unplanned and prolonged ambivalent	0.48 (0.27; 0.71)	0.42 (0.15; 0.72)	0.06 (-0.16; 0.28)	0.38 (0.19-0.57)	0.39 (0.14; 0.66)	-0.02 (-0.21; 0.18)
**Age 3 years**						
Unplanned and wanted	0.16 (0.10-0.22)	0.14 (0.07; 0.22)	0.01 (-0.03; 0.06)	0.15 (0.09-0.21)	0.14 (0.06; 0.21)	0.01 (-0.04; 0.06)
Unplanned and initially ambivalent	0.24 (0.17-0.32)	0.24 (0.15; 0.33)	0.00 (-0.06; 0.07)	0.22 (0.15-0.29)	0.23 (0.14; 0.32)	-0.01 (-0.07; 0.05)
Unplanned and prolonged ambivalent	0.65 (0.43-0.88)	0.66 (0.38; 0.97)	-0.02 (-0.25; 0.21)	0.56 (0.37-0.77)	0.61 (0.34; 0.89)	-0.05 (-0.26; 0.16)
**Age 6 years**						
Unplanned and wanted	0.13 (0.07; 0.20)	0.12 (0.05; 0.20)	0.01 (-0.04; 0.06)	0.16 (0.10; 0.22)	0.15 (0.08; 0.22)	0.01 (-0.04; 0.06)
Unplanned and initially ambivalent	0.22 (0.15; 0.30)	0.21 (0.12; 0.31)	0.01 (-0.05; 0.08)	0.20 (0.13; 0.27)	0.19 (0.10; 0.29)	0.01 (-0.06; 0.07)
Unplanned and prolonged ambivalent	0.30 (0.10; 0.51)	0.30 (0.04; 0.58)	0.00 (-0.21; 0.21)	0.34 (0.14; 0.54)	0.33 (0.07; 0.60)	0.01 (-0.19; 0.22)
**Age 9 years**						
Unplanned and wanted	0.12 (0.06; 0.18)	0.13 (0.05; 0.20)	0.00 (-0.05; 0.04)	0.13 (0.07; 0.19)	0.14 (0.07; 0.21)	-0.01 (-0.06; 0.04)
Unplanned and initially ambivalent	0.20 (0.13; 0.28)	0.21 (0.11; 0.30)	0.00 (-0.07; 0.06)	0.27 (0.20; 0.35)	0.27 (0.17; 0.37)	0.00 (-0.06; 0.04)
Unplanned and prolonged ambivalent	0.52 (0.30; 0.74)	0.49 (0.20; 0.81)	0.02 (-0.20; 0.26)	0.48 (0.27; 0.71)	0.51 (0.21; 0.83)	-0.03 (-0.26; 0.20)
**Age 13 years**						
Unplanned and wanted	0.14 (0.08; 0.20)	0.13 (0.06; 0.20)	0.00 (-0.04; 0.06)	0.15 (0.09; 0.22)	0.15 (0.08; 0.23)	0.00 (-0.05; 0.05)
Unplanned and initially ambivalent	0.21 (0.14; 0.28)	0.20 (0.11; 0.30)	0.01 (-0.06; 0.07)	0.22 (0.15; 0.29)	0.20 (0.11; 0.30)	0.02 (-0.05; 0.08)
Unplanned and prolonged ambivalent	0.33 (0.14; 0.54)	0.30 (0.03; 0.60)	0.03 (-0.18; 0.24)	0.37 (0.17; 0.59)	0.38 (0.09; 0.69)	0.00 (-0.23; 0.22)

* In women who participated in the study with multiple pregnancies or who gave birth to twins, a random child was excluded, leaving N=8892 participants.

Before: The absolute difference in internalizing and externalizing problem behaviour (beta-coefficients) between the intention groups with planned pregnancy as reference and 95% confidence interval in between brackets.

After increasing social support: The absolute difference in internalizing and externalizing problem behaviour (beta-coefficients) between the intention groups with planned pregnancy as reference and 95% confidence interval in between brackets after setting the study population to 'sufficient social support'.

Reduction: The absolute change of the beta-coefficients without and with intervention in internalizing and externalizing problem behaviour between the intention groups with planned pregnancy as reference and 95% confidence interval in between brackets.

The 95% confidence intervals were calculated by bootstrapping with 1000 iterations.

Table S6: Changes in behavior differences by hypothetically eliminating postnatal depression using complete cases* only.

	Internalizing behavior			Externalizing behavior		
	Before	After eliminating postnatal depression	Reduction	Before	After eliminating postnatal depression	Reduction
**Age 1.5 years**						
Unplanned and wanted	0.13 (0.04; 0.21)	0.10 (0.02; 0.18)	0.03 (7.5^{e-4}; 0.07)*	0.10 (0.01-0.20)	0.07 (0.02; 0.17)	0.03 (-0.01; 0.07)
Unplanned and initially ambivalent	0.20 (0.10; 0.31)	0.18 (0.08; 0.28)	0.03 (-0.02; 0.08)	0.20 (0.08-0.32)	0.21 (0.08; 0.35)	-0.01 (-0.06; 0.04)
Unplanned and prolonged ambivalent	0.25 (0.02; 0.52)	0.32 (0.04; 0.63)	-0.06 (-0.20; 0.06)	0.31 (0.03-0.60)	0.30 (0.01; 0.61)	0.01 (-0.16; 0.19)
**Age 3 years**						
Unplanned and wanted	0.10 (0.02; 0.19)	0.08 (0.01; 0.16)	0.02 (-0.02; 0.06)	0.12 (0.03-0.21)	0.10 (0.01; 0.19)	0.01 (-0.02; 0.05)
Unplanned and initially ambivalent	0.15 (0.05; 0.26)	0.16 (0.06; 0.27)	-0.01 (-0.06; 0.04)	0.16 (0.05-0.28)	0.18 (0.05; 0.30)	-0.01 (-0.06; 0.03)
Unplanned and prolonged ambivalent	0.43 (0.18; 0.72)	0.48 (0.19; 0.81)	-0.05 (-0.21; 0.11)	0.49 (0.20-0.80)	0.49 (0.18; 0.82)	0.00 (-0.19; 0.20)
**Age 6 years**						
Unplanned and wanted	0.08 (3.7^{e-3}; 0.17)	0.07 (-0.01; 0.15)	0.01 (-0.02; 0.05)	0.13 (0.05-0.22)	0.13 (0.04; 0.21)	0.01 (-0.02; 0.04)
Unplanned and initially ambivalent	0.13 (0.02; 0.25)	0.12 (0.01; 0.23)	0.01 (-0.04; 0.07)	0.14 (0.02-0.25)	0.12 (-2.6^{e-3}; 0.24)	0.01 (-0.03; 0.06)
Unplanned and prolonged ambivalent	0.10 (-0.14; 0.35)	0.13 (-0.11; 0.39)	-0.03 (-0.19; 0.14)	0.27 (-0.02-0.58)	0.30 (0.01; 0.60)	-0.03 (-0.22; 0.18)
**Age 9 years**						
Unplanned and wanted	0.07 (-0.02; 0.15)	0.05 (-0.04; 0.13)	0.02 (-0.01; 0.06)	0.13 (0.04; 0.22)	0.11 (0.02; 0.20)	0.02 (-0.02; 0.06)
Unplanned and initially ambivalent	0.19 (-0.06; 0.32)	0.16 (0.03; 0.30)	0.03 (-0.03; 0.09)	0.23 (0.11; 0.36)	0.25 (0.12; 0.39)	-0.02 (-0.07; 0.03)
Unplanned and prolonged ambivalent	0.45 (0.13; 0.79)	0.38 (0.08; 0.71)	0.07 (-0.14; 0.30)	0.46 (0.16; 0.79)	0.49 (0.17; 0.83)	-0.03 (-0.21; 0.18)
**Age 13 years**						
Unplanned and wanted	0.13 (0.03; 0.23)	0.11 (0.01; 0.21)	0.02 (-0.02; 0.06)	0.11 (0.03; 0.20)	0.10 (0.01; 0.19)	0.02 (-0.02; 0.06)
Unplanned and initially ambivalent	0.18 (0.06; 0.31)	0.15 (0.03; 0.28)	0.03 (-0.02; 0.09)	0.17 (0.05; 0.30)	0.20 (0.07; 0.33)	-0.03 (-0.07; 0.02)
Unplanned and prolonged ambivalent	0.22 (-0.04; 0.51)	0.20 (-0.08; 0.52)	0.03 (-0.13; 0.20)	0.26 (-0.01; 0.56)	0.29 (0.01; 0.62)	0.04 (-0.20; 0.14)

* Significant reduction. The 95% confidence intervals were calculated by bootstrapping with 1000 iterations.

**Dyads with missing data on unintended pregnancy, postnatal depression or all child behavior questionnaires were excluded, leaving N=4034 participants.

Before: The absolute difference in internalizing and externalizing problem behaviour (beta-coefficients) between the intention groups with planned pregnancy as reference and 95% confidence interval in between brackets.

After eliminating postnatal depression: The absolute difference in internalizing and externalizing problem behaviour (beta-coefficients) between the intention groups with planned pregnancy as reference and 95% confidence interval in between brackets after setting the study population to ‘no postnatal depression’.

Reduction: The absolute change of the beta-coefficients without and with intervention in internalizing and externalizing problem behaviour between the intention groups with planned pregnancy as reference and 95% confidence interval in between brackets.

Table S7: Changes in behavior differences by hypothetically increasing social support using complete cases* only.

	Internalizing behavior			Externalizing behavior		
	**Before**	**After increasing social support**	**Reduction**	**Before**	**After increasing social support**	**Reduction**
**Age 1.5 years**						
Unplanned and wanted	0.16 (0.07; 0.25)	0.16 (0.05; 0.27)	0.00 (-0.06; 0.07)	0.15 (0.05-0.26)	0.14 (0.02; 0.27)	0.01 (-0.04; 0.06)
Unplanned and initially ambivalent	0.27 (0.15; 0.41)	0.27 (0.13; 0.42)	0.00 (-0.10; 0.12)	0.22 (0.09-0.36)	0.31 (0.15; 0.48)	-0.09 (-0.07; 0.05)
Unplanned and prolonged ambivalent	0.25 (1.6^{e-3}; 0.53)	0.25 (-0.05; 0.73)	0.00 (-0.25; 0.26)	0.15 (-0.10-0.42)	0.36 (0.02; 0.70)	-0.21 (-0.46; 0.03)
**Age 3 years**						
Unplanned and wanted	0.18 (0.09; 0.28)	0.16 (0.05; 0.27)	0.02 (-0.05; 0.09)	0.19 (0.09-0.30)	0.17 (0.05; 0.30)	0.02 (-0.06; 0.09)
Unplanned and initially ambivalent	0.21 (0.09; 0.34)	0.24 (0.09; 0.40)	-0.03 (-0.12; 0.07)	0.22 (0.09; 0.36)	0.31 (0.14; 0.47)	-0.08 (-0.19; 0.02)
Unplanned and prolonged ambivalent	0.34 (0.10; 0.60)	0.45 (0.13; 0.81)	-0.11 (-0.37; 0.14)	0.33 (0.04-0.64)	0.42 (0.02; 0.83)	-0.09 (-0.39; 0.21)
**Age 6 years**						
Unplanned and wanted	0.18 (0.08; 0.18)	0.17 (0.05; 0.28)	0.01 (-0.06; 0.09)	0.22 (0.12-0.32)	0.21 (0.10; 0.33)	0.01 (-0.07; 0.08)
Unplanned and initially ambivalent	0.21 (0.08; 0.34)	0.21 (0.05; 0.39)	0.00 (-0.11; 0.10)	0.21 (0.08-0.34)	0.24 (0.08; 0.41)	-0.03 (-0.14; 0.07)
Unplanned and prolonged ambivalent	0.03 (-0.22; 0.30)	0.18 (-0.19; 0.59)	-0.15 (-0.43; 0.11)	0.08 (-0.20-0.38)	0.17 (-0.16; 0.52)	-0.09 (-0.36; 0.18)
**Age 9 years**						
Unplanned and wanted	0.16 (0.06; 0.27)	0.18 (0.05; 0.31)	-0.01 (-0.09; 0.07)	0.19 (0.08; 0.30)	0.24 (0.11; 0.38)	-0.05 (-0.13; 0.03)
Unplanned and initially ambivalent	0.20 (0.06; 0.35)	0.23 (0.06; 0.42)	-0.03 (-0.15; 0.09)	0.24 (0.10; 0.39)	0.30 (0.12; 0.50)	-0.06 (-0.18; 0.05)
Unplanned and prolonged ambivalent	0.32 (-4.6^{e-3}; 0.66)	0.41 (-0.05; 0.92)	-0.09 (-0.42; 0.22)	0.28 (-0.03; 0.62)	0.39 (-0.05; 0.86)	-0.11 (-0.42; 0.20)
**Age 13 years**						
Unplanned and wanted	0.18 (0.07; 0.29)	0.16 (0.04; 0.29)	0.01 (-0.07; 0.09)	0.16 (0.06; 0.26)	0.18 (0.06; 0.31)	-0.02 (-0.10; 0.06)
Unplanned and initially ambivalent	0.20 (0.07; 0.34)	0.21 (0.04; 0.39)	-0.01 (-0.12; 0.11)	0.19 (0.06; 0.32)	0.21 (0.05; 0.39)	-0.01 (-0.13; 0.09)
Unplanned and prolonged ambivalent	0.22 (-0.07; 0.53)	0.23 (0.19; 0.71)	-0.01 (-0.31; 0.28)	0.19 (-0.07; 0.49)	0.28 (-0.12; 0.73)	-0.09 (-0.37; 0.18)

*Dyads with missing data on unintended pregnancy, social support or all child behavior questionnaires were excluded, leaving N=3484 participants.

Before: The absolute difference in internalizing and externalizing problem behaviour (beta-coefficients) between the intention groups with planned pregnancy as reference and 95% confidence interval in between brackets.

After increasing social support: The absolute difference in internalizing and externalizing problem behaviour (beta-coefficients) between the intention groups with planned pregnancy as reference and 95% confidence interval in between brackets after setting the study population to ‘sufficient social support’.

Reduction: The absolute change of the beta-coefficients without and with intervention in internalizing and externalizing problem behaviour between the intention groups with planned pregnancy as reference and 95% confidence interval in between brackets.

The 95% confidence intervals were calculated by bootstrapping with 1000 iterations.

Table S8: Changes in borderline clinical cases, by hypothetically eliminating postnatal depression.

	**Internalizing behavior**			**Externalizing behavior**		
	**Before**	**After eliminating postnatal depression**	**Reduction**	**Before**	**After eliminating postnatal depression**	**Reduction**
**Age 1.5 years**						
Unplanned and wanted	4.8% (2.9%; 6.7%)	3.7% (1.7%; 5.8%)	-1.0% (-2.2%; 0.0%)*	4.8% (2.6%; 7.0%)	4.1% (1.8%; 6.4%)	-0.7% (-1.8%; 0.4%)
Unplanned and initially ambivalent	8.4% (6.1%; 10.8%)	6.1% (3.6%; 8.7%)	-2.4% (-4.0%; -0.8%)*	8.3% (5.8%; 11.0%)	6.8% (4.0%; 9.8%)	-1.5% (-3.1%; 0.1%)
Unplanned and prolonged ambivalent	12.1% (5.6%; 18.9%)	9.1% (1.1%; 17.6%)	-3.0% (-9.0%; 2.7%)	12.5% (5.7%; 19.9%)	9.1% (0.7%; 17.8%)	-3.5% (-9.5%; 2.2%)
**Age 3 years**						
Unplanned and wanted	4.0% (2.1%; 5.9%)	3.2% (1.2%; 5.2%)	-0.8% (-1.9%; 0.2%)	3.8% (2.0%; 5.6%)	3.2% (1.4%; 5.1%)	-0.5% (-1.5%; 0.4%)
Unplanned and initially ambivalent	6.9% (4.6%; 9.3%)	6.0% (3.4%; 8.6%)	-1.0% (-2.5%; 0.5%)	6.4% (4.3%; 8.6%)	5.3% (2.9%; 7.7%)	-1.1% (-2.5%; 0.3%)
Unplanned and prolonged ambivalent	17.2% 10.3%; 24.5%)	15.5% (6.6%; 24.8%)	-1.7% (-8.0%; 4.3%)	15.1% (8.6%; 22.0%)	12.9% (4.8%; 21.6%)	-2.2% (-8.4%; 3.5%)
**Age 6 years**						
Unplanned and wanted	4.2% (2.2%; 6.3%)	3.4% (1.3%; 5.6%)	-0.8% (-2.0%; 0.3%)	3.2% (1.5%; 4.9%)	2.4% (0.7%; 4.1%)	-0.8% (-1.8%; 0.1%)
Unplanned and initially ambivalent	6.6% (4.2%; 9.0%)	4.5% (1.9%; 7.2%)	-2.1% (-3.7%; -0.5%)*	5.3% (3.2%; 7.4%)	3.9% (1.7%; 6.1%)	-1.4% (-2.8%; -0.1%)*
Unplanned and prolonged ambivalent	9.6% (3.2%; 16.4%)	4.7% (-2.6%; 12.8%)	-4.9% (-11.0%; 0.9%)	6.0% (0.9%; 11.7%)	2.3% (-3.2%; 8.6%)	-3.7% (-8.8%; 0.9%)
**Age 9 years**						
Unplanned and wanted	3.7% (1.6%; 5.9%)	3.1% (0.9%; 5.4%)	-0.6% (-1.7%; 0.5%)	3.4% (1.7%; 5.2%)	2.9% (1.1%; 4.7%)	-0.5% (-1.5%; 0.4%)
Unplanned and initially ambivalent	6.9% (4.4%; 9.4%)	5.6% (2.7%; 8.5%)	-1.3% (-2.9%; 0.3%)	6.5% (4.3%; 8.7%)	5.5% (3.1%; 7.9%)	-1.0% (-2.4%; 0.4%)
Unplanned and prolonged ambivalent	16.9% (9.6%; 24.5%)	13.5% (4.3%; 22.9%)	-3.4% (-9.8%; 2.5%)	12.6% (6.3%; 19.2%)	10.5% (2.9%; 18.8%)	-2.1% (-7.8%; 3.3%)
**Age 13 years**						
Unplanned and wanted	4.0% (1.9%; 6.3%)	3.5% (1.2%; 5.9%)	-0.5% (-1.6%; 0.6%)	3.8% (2.0%; 5.6%)	3.2% (1.4%; 5.1%)	-0.6% (-1.5%; 0.4%)
Unplanned and initially ambivalent	6.4% (3.9%; 9.0%)	4.5% (1.7%; 7.4%)	-1.9% (-3.6%; -0.3%)*	5.8% (3.7%; 7.9%)	5.2% (2.8%; 7.7%)	-0.5% (-1.9%; 0.8%)
Unplanned and prolonged ambivalent	12.1% (5.1%; 19.2%)	7.8% (-0.6%; 16.7%)	-4.3% (-10.5%; 1.6%)	8.9% (3.2%; 15.1%)	5.6% (-1.0%; 12.9%)	-3.3% (-8.7%; 1.7%)

* Significant reduction. The 95% confidence intervals were calculated by bootstrapping with 1000 iterations.

Before: The absolute difference in proportions of borderline clinical cases in internalizing and externalizing problem behaviour between the intention groups with planned pregnancy as reference and 95% confidence interval in between brackets.

After eliminating postnatal depression: The absolute difference in proportions of borderline clinical cases in internalizing and externalizing problem behaviour between the intention groups with planned pregnancy as reference and 95% confidence interval in between brackets after setting the study population to ‘no postnatal depression’.

Reduction: The absolute change of the differences in proportions without and with intervention in proportions of borderline clinical cases in internalizing and externalizing problem behaviour between the intention groups with planned pregnancy as reference and 95% confidence interval in between brackets.
